# Response of microbial structure characteristics and enzyme activity to different altitude

**DOI:** 10.3389/fmicb.2025.1588591

**Published:** 2025-04-23

**Authors:** Qiu-liang Cai, Gui-kang Jia, Chun Wei, Ning Zhong, Ling-ling Lv, Jian Li, Hong Pang, Wei Yang

**Affiliations:** ^1^Guangxi Key Laboratory of Biology for Mango, Agriculture and Food Engineering College, Industrial College of Subtropical Characteristic Agriculture, Baise University, Baise, China; ^2^College of Chemistry, Chemical Engineering & Environmental Science, Minnan Normal University Minnan Normal University, Zhangzhou, China

**Keywords:** elevation, soil, enzymatic activity, soil microbial diversity, redundancy analysis

## Abstract

Studying the influence of altitude on soil microorganisms and enzyme activity is crucial for protecting land environmental changes in karst areas. The study focused on the soil at different altitudes in the Luganyou area, Baise City. The Multidimensional statistical analysis encompassed assessments of soil nutrient properties, soil microorganisms, and soil enzymes across these altitude gradients. The results indicate that enzyme activity in the 0–15 cm soil layer surpasses that in the 15–30 cm soil layer. Catalase, urease, and soil cellulase exhibit consistent trends on both shady and sunny slopes, increasing with altitude. Alpha diversity analysis shows that the abundance and diversity of bacteria and fungi on the shaded side exhibit the following trend of low altitude > high altitude. In contrast, on the sunny side, bacterial richness displayed a decreasing-increasing pattern with rising altitude, while the diversity trend mirrored that of the shady side. Overall, fungal richness on the sunny side exhibited a slight decrease, whereas diversity increased with altitude. Across shady slopes, overall soil nutrient levels tend to increase with altitude. The comprehensive ecological stoichiometry analysis of the soil indicated an elevated C/N ratio, while the N/P and C/P ratios were relatively low on both shady and sunny slopes. These findings suggest that the study area as a whole is constrained by N availability. Correlation and redundancy analyses revealed that organic matter is the primary factor influencing enzyme activity in shaded slopes, while organic matter and available phosphorus are the key factors affecting enzyme activity in sunny slopes. The key nutrient factors affecting microorganisms include total nitrogen content, organic matter content, as well as enzyme factors such as invertase, catalase, and acid phosphatase. In summary, the study revealed a negative correlation between microbial diversity on shady slopes and altitude in the low altitude areas of southwest China (160–380 m). Additionally, bacterial richness on sunny slopes displayed a “low-high-low” trend, underscoring the significance of organic matter (*R*^2^ = 0.683) and available phosphorus (*p* < 0.05) as pivotal drivers of microbial communities. These findings aim to offer a theoretical framework for guiding crop cultivation, land management, and enhancement strategies in southwestern China.

## 1 Introduction

Terrestrial ecosystems are important places for life-sustaining activities. Soil microorganisms serve as an important driving force and link in maintaining terrestrial ecosystems (Guan, 1986; Dietert and Dietert, 2024), representing the dynamic and highly active component of soil (Holguin et al., 2001). Environmental factors affect the number and community structure of soil microorganisms. Altitude and tropism indirectly influence soil microorganisms by regulating microclimate, vegetation type, soil characteristics, and other factors, thereby impacting soil light, heat, water, air and other conditions. These influences lead to alterations in the physical, chemical, and biological attributes of the soil, consequently affecting soil nutrients, enzyme activity, and microbial diversity either directly or indirectly (Kumar and Garkoti, 2022; Zhang et al., 2025). Soil enzymes primarily originate from plant root exudates, soil microorganism activities, and the decomposition of plant and animal residues (Jacobson Meyers et al., 2014). The soil enzyme activity can reflect changes in soil physical and chemical properties, biomass, and biodiversity, and can also promote changes in soil ecosystems. The cycle of carbon (C), nitrogen (N), phosphorus (P), and other nutrients (Sainju et al., 2022; Shah et al., 2024) is a significant driver of soil ecosystem metabolism (Delgado-Baquerizo, 2019). And the activity of soil enzymes serves as a valuable indicator of soil fertility levels, representing a key metric for assessing soil fertility status (Obayomi et al., 2023).

Previous studies have demonstrated a notable decrease in the taxonomic diversity of soil bacteria and fungi with increasing altitude (Schinner, 1982; Liu, 2010; Fuentes et al., 2022). Fuentes et al. (2022) reported reduced bacterial and fungal diversity at higher altitudes in Mediterranean ecosystems, attributed to harsh environmental conditions. Wan et al. (2021) observed a non-linear trend in the activity of β-glucosidase (βG) and other enzymes in Helan Mountain soil. These findings collectively indicate that altitude variations influence the community structure, enzyme activity, and nutrient status of soil bacteria and other microorganisms.

However, most research to date has focused on single-factor effects (e.g., enzyme activity or microbial diversity) in high-elevation regions, leading to gaps in understanding the integrated responses of soil enzymes, nutrients, and microbial communities in low-elevation karst ecosystems of Southwest China. This study addresses this critical gap by investigating the interactive effects of altitude and slope aspect on soil nutrients, enzyme activities, and microbial communities in the Luganyou Mountain region. This area, located in Guangxi, China, represents a typical low-altitude (160–380 m) karst ecosystem surrounded by mango trees. The high-throughput sequencing and structural equation modeling were employed to explore the multivariate relationships among soil properties, enzymes, and microbial diversity. The analysis aimed to elucidate the key environmental factors influencing these relationships, with the goal of providing a theoretical framework and data repository for understanding the mechanisms governing soil processes in low-altitude karst environments and promoting the sustainable utilization of biological resources in southwestern China.

## 2 Materials and methods

### 2.1 Study area

The study area, Baise City, is situated at the confluence of the Yunnan-Guizhou Plateau and the hilly regions of Guangxi. Positioned at low latitudes, it experiences a characteristic subtropical monsoon climate characterized by extended summers and brief winters, high humidity, comparable spring and autumn seasons, abundant sunshine, adequate warmth, and moderate and concentrated rainfall (Wang et al., 2024). Notably, the months from June to August receive a substantial share of the annual precipitation, contributing approximately 75% of the total yearly rainfall. The region experiences an average annual precipitation of 1,115 mm, with an average annual temperature around 22.1°C. The topography of the Youjiang District features elevated terrain in the northern and southern areas, gradually descending toward the central part. Characterized by rugged and steep landscapes, the area is marked by continuous mountain ranges and deep valleys. Altitudes in the region typically range from 150 to 1,500 m above sea level (Meng et al., 2022). This study mainly focuses on the ecological environment of mangoes at different altitudes. The altitude range of mango planting areas is mostly concentrated between 150 and 380 m. Therefore, to understand the ecological environment of mangoes, the altitude range of 150–380 m has been chosen for the research.

### 2.2 Soil samples collection

In this study, the sampling area is surrounded by mango trees. The soil samples were collected at different elevations along the shady and sunny slopes of Luganyou Mountain in Baise City, spanning from low to high altitudes. Six sampling points were designated on both the shady and sunny slopes, totaling 24 soil samples once in the winter of 2022. The sampling details are outlined in Supplementary Table 1. Stringent sterility measures were implemented to ensure the scientific rigor and reliability of the samples. Each soil sample collection involved the sterilization of sampling tools, removal of surface debris using a shovel, and employing a five-point sampling technique using a soil auger to extract soil samples from depths of 0–15 cm and 15–30 cm. The soil samples were meticulously deposited into properly labeled sterile sampling bags, each weighing approximately 150 g, for microbial cultivation purposes. Additionally, separate soil samples weighing about 150 g each were collected for the evaluation of soil physical and chemical properties, as well as soil enzyme activity. Stringent measures were observed during sampling, with researchers donning disposable nitrile gloves and masks to mitigate any risk of soil sample cross-contamination. Following collection, all soil samples were transferred into cryogenic storage boxes and promptly transported back to the laboratory for refrigeration.

### 2.3 Soil nutrient properties

Soil nutrient characteristics are mainly influenced by indicators such as total nitrogen, total phosphorus, total potassium, nitrate nitrogen, ammonium nitrogen, available phosphorus, available potassium, organic matter, and pH. Soil total nitrogen (TN) was determined by a semi-micro Kelvin method, while soil total phosphorus (TP) was determined by the sulfuric acid-perchloric acid boiling method. Ammonium nitrogen (NH^4+^-N) was determined by the Nessler reagent photometric method, and nitrate nitrogen (NO_3_^−^-N) was determined by ultraviolet spectrophotometry. Molybdenum antimony resistance spectrophotometry was used to estimate available phosphorus (Yokamo et al., 2023; Wang and Cai, 2024; Xie, 2014; Guan and Meng, 1986). Each soil sample underwent processing in triplicate, and average values were subsequently calculated.

### 2.4 Soil enzyme activity

Cellulase activity was determined by the 3,5-dinitrosalicylic acid colorimetric method, with results reported as mg of glucose produced by 1 g of dry soil at 72 h. Soil invertase activity was also determined by the 3,5-dinitrosalicylic acid colorimetric method and expressed as mg of glucose produced by 1 g of dry soil at 24 h. Catalase activity was determined using the titration method, measuring the volume of potassium permanganate solution (0.1 mol/L) consumed by 2 g of soil. Soil urease activity was determined by indophenol blue colorimetry and expressed as mg of ammonia-nitrogen produced by 1 g of soil at 37°C for 24 h. Acid phosphatase activity was determined by the 1,4-dinitrophenyl phosphate method, measuring the mass of phenol released by 1 g of soil after 24 h (Wu, 2006). Each soil sample underwent triplicate analyses, with average values subsequently calculated.

### 2.5 Soil microbial groups quantification

Soil microorganism counts was determined by the plate count method (Liu et al., 2016). Soil bacteria were grown on nutrient agar (Beef extract 5.0 g, peptone 10.0 g, NaCl 5.0 g, distilled H_2_O, 1,000 mL, agar 20 g, pH 7.2–7.4). Soil fungi were cultured in Martin-Bengal red agar medium (soluble starch 20 g, KNO_3_ 1 g, K_2_HPO_4_ 0.5 g, MgSO_4_ 7H_2_0 0.5 g, NaCl 0.5 g, FeSO_4_ 7H2O 0.01 g, pH7.2–7.4). Actinomycetes were enumerated using Medium No. 1 (glucose 10.0 g, MgSO_4_·7H2O 0.5 g, peptone 5.0 g, Bengal red 33.4 mg, K_2_HPO_4_ 1 g, distilled water H_2_0 1,000 mL, pH 4–5).

### 2.6 Illumina MiSeq sequencing

Purified amplicons were pooled in equimolar amounts and paired-end sequenced on an Illumina MiSeq PE300 platform/NovaSeq PE250 platform (Illumina, San Diego, United States), according to the standard protocols of Majorbio Bio-Pharm Technology Co. Ltd. (Shanghai, China).

### 2.7 Data processing and statistical analysis

Raw FASTQ files were de-multiplexed utilizing a custom perl script and subsequently subjected to quality filtration using fastp version 0.19.6 (Chen et al., 2018). Subsequently, the sequences were merged using FLASH version 1.2.7 (Magoč and Salzberg, 2011).

Soil enzyme activities, soil microbial quantities, and soil physical and chemical characteristics at various altitudes were statistically analyzed, organized, and visualized using Microsoft Excel 2020 worksheets and Origin2022 software for plotting. Significance testing, variance analysis, correlation analysis, redundancy analysis, and principal component analysis of soil nutrients, enzyme activities, and microorganisms were conducted using IBM SPSS-22 statistical software. Additionally, Data redundancy analysis (RDA) using canoco 5 software.

## 3 Results

### 3.1 Changes in soil enzyme activity at different altitudes

**Figure 1:**
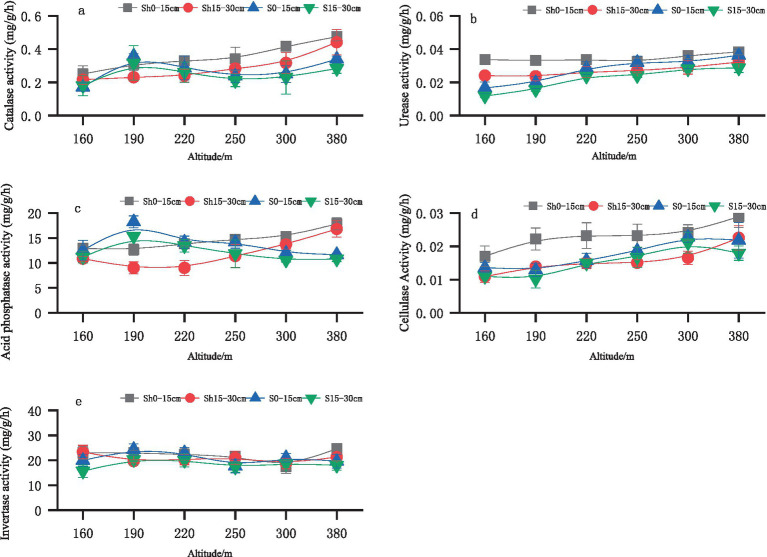
Changes in catalase **(a)**, urease **(b)**, acid phosphatase **(c)**, cellulase **(d)** and invertase **(e)** activities in soils.

As depicted in Figure 1a, the collective catalase activity on shady slopes exhibits an ascending trend with rising altitude, with the overall catalase activity in the 0–15 cm soil layer surpassing that in the 15–30 cm soil layer. The catalase activity peaked at 380 m above sea level, registering at 0.48 mg/g/h for the 0–15 cm layer and 0.44 mg/g/h for the 15–30 cm layer, respectively. Conversely, on the sunny slope, the collective catalase activity displayed a pattern of initial slight increase, followed by a decrease and subsequent increase. Comparatively, the catalase activity in the 0–15 cm soil layer tended to be higher than that in the 15–30 cm soil layer, with peak values observed at an altitude of 190 m, measuring 0.36 mg/g/h and 0.32 mg/g/h, respectively.

In Figure 1b, it is evident that the urease activity in the 0–15 cm soil layer is notably higher than that in the 15–30 cm soil layer across varying altitudes. Urease activity on the shady slope demonstrates an increasing trend with altitude, while on sunny slopes, the rise in urease activity is markedly more pronounced compared to shady slopes. The peak urease activity for both shady and sunny slopes is observed at 380 meters above sea level. Specifically, the urease activity on shady slopes is recorded as 0.038 mg/g/h for the 0–15 cm layer and 0.032 mg/g/h for the 15–30 cm layer, whereas on sunny slopes, it is measured as 0.036 mg/g/h for the 0–15 cm layer and 0.029 mg/g/h for the 15–30 cm layer.

The acid phosphatase activity in the 0–15 cm soil layer surpasses that in the 15–30 cm soil layer (Figure 1c). Acid phosphatase activity on the shady slope exhibits a steady ascending trend, reaching its peak at 380 m above sea level, with values of 17.86 mg/g/h for the 0–15 cm layer and 16.82 mg/g/h for the 15–30 cm layer. Conversely, the overall pattern on sunny slopes shows an initial increase followed by a subsequent decrease in acid phosphatase activity.

In Figure 1d, it is evident that the cellulase activity in the 0–15 cm soil layer exceeds that in the 15–30 cm soil layer across altitudes. The overall trend indicates an increase with altitude. The peak cellulase activity was achieved at 380 m above sea level, with values of 0.029 mg/g/h for the 0–15 cm layer and 0.023 mg/g/h for the 15–30 cm layer. On sunny slopes, cellulase activity escalates with altitude, reaching its zenith at 300 m above sea level, registering as 0.023 mg/g/h for the 0–15 cm layer and 0.021 mg/g/h for the 15–30 cm layer; however, the difference from the peak at 380 m is not significant.

No significant disparity is observed in the distribution and activity of soil invertase enzymes across the shady and sunny soil layers on the slopes, as depicted in Figure 1e.

### 3.2 Illumina MiSeq sequencing results

The quality control assessment of high-throughput sequencing data revealed that a total of 549,167 valid bacterial sequences and 640,343 valid fungal sequences were obtained across the 12 sample groups (Supplementary Figure 1). Rarefaction analysis indicated that the saturation points of the dilution curves for each sample plateaued, indicating adequate data acquisition that accurately portrays the community composition of soil bacteria and fungi in each sample.

### 3.3 OTUs cluster analysis

The total number of OTUs for bacterial communities across the 12 groups is 577, indicating the detection of 577 bacterial species at varying altitudes (Supplementary Figure 2a). In Supplementary Figure 2b, there are 10 OTUs shared among various fungal groups. Significant variations are observed in the bacterial species composition at different altitudes, with Group 2 exhibiting the highest number of unique bacterial OTUs at 147. In contrast, fungi display minimal differences in unique OTUs across the groups.

### 3.4 Alpha diversity analysis

The Alpha diversity indices of the 24 samples, as presented in Table 1, indicate that the ACE index for soil bacteria ranges from 1869.777 to 38.02.338, while the Chao index ranges from 1831.619 to 3846.903. The Shannon index varies between 5.352 and 6.656, and the Simpson index ranges from 0.003 to 0.014. In the shady slope samples, there is a diminishing trend in bacterial richness and diversity as altitude increases. Conversely, on sunny slopes, bacterial richness initially decreases and then shows a slight increase with altitude, mirroring the diversity trend observed on shady slopes. The ACE index for fungi ranges from 445.195 to 1290.721, with the Chao index varying between 415.593 and 1289.975. The Shannon index for fungi ranges from 2.380 to 5.352, and the Simpson index ranges from 0.020 to 0.303. In the samples from shady slopes, the richness and diversity of fungi decrease with increasing altitude. Conversely, on sunny slopes, there is a slight decrease in overall fungal richness, while diversity shows an increasing trend with altitude.

**Table 1:** Statistics of α-diversity index of each sample group.

Sample	Ace	Chao	Shannon	Simpson	Coverage
Bacteria					
Sh160 0-15	3054.264	3060.822	6.621	0.003	0.989
Sh160 15-30	2866.912	2902.405	6.591	0.003	0.987
Sh190 0-15	3251.482	3283.686	6.318	0.007	0.988
Sh190 15-30	2578.033	2626.225	5.965	0.007	0.987
Sh220 0-15	3059.110	3129.640	6.343	0.006	0.987
Sh220 15-30	3389.173	3344.460	6.543	0.005	0.985
Sh250 0-15	2357.536	2301.612	5.671	0.010	0.990
Sh250 15-30	2026.305	2046.686	5.503	0.011	0.991
Sh300 0-15	1948.162	1922.961	5.528	0.009	0.993
Sh300 15-30	2013.619	1954.037	5.610	0.008	0.991
Sh380 0-15	2733.993	2680.250	5.867	0.007	0.992
Sh380 15-30	2298.380	2288.421	5.574	0.008	0.989
S160 0-15	3802.338	3846.903	6.656	0.004	0.985
S160 15-30	3661.776	3615.636	6.481	0.005	0.983
S190 0-15	1961.913	1976.005	5.649	0.008	0.992
S190 15-30	1993.791	1994.299	5.576	0.010	0.991
S220 0-15	1915.722	1938.107	5.566	0.009	0.994
S220 15-30	1949.282	1894.655	5.650	0.008	0.993
S250 0-15	2267.636	2254.989	5.865	0.007	0.993
S250 15-30	2420.346	2424.595	5.693	0.009	0.990
S300 0-15	2138.148	2115.506	5.692	0.008	0.991
S300 15-30	1869.777	1831.619	5.478	0.012	0.992
S380 0-15	2819.031	2859.446	5.798	0.009	0.988
S380 15-30	2468.714	2414.087	5.352	0.014	0.990
Fungus					
Sh160 0-15	1290.721	1289.975	5.037	0.020	0.999
Sh160 15-30	877.414	888.098	4.122	0.068	0.999
Sh190 0-15	954.770	952.826	4.189	0.040	0.998
Sh190 15-30	518.267	534.393	2.970	0.119	0.998
Sh220 0-15	886.744	883.143	3.131	0.164	0.998
Sh220 15-30	1082.186	1079.904	4.244	0.050	0.997
Sh250 0-15	816.803	819.014	4.232	0.050	0.999
Sh250 15-30	716.801	715.448	3.317	0.099	0.998
Sh300 0-15	445.195	415.593	2.479	0.160	0.998
Sh300 15-30	579.216	569.130	2.915	0.127	0.998
Sh380 0-15	677.851	668.140	2.380	0.303	0.998
Sh380 15-30	576.190	564.217	3.104	0.117	0.998
S160 0-15	932.394	908.342	2.768	0.159	0.997
S160 15-30	763.608	761.777	2.619	0.147	0.997
S190 0-15	670.599	646.042	3.123	0.092	0.998
S190 15-30	687.162	689.75	3.461	0.066	0.998
S220 0-15	811.000	806.532	3.941	0.048	0.998
S220 15-30	731.965	738.243	4.265	0.033	0.998
S250 0-15	799.650	796.125	4.238	0.035	0.998
S250 15-30	791.270	798.570	3.932	0.052	0.998
S300 0-15	890.137	872.598	3.393	0.159	0.998
S300 15-30	602.715	617.702	2.931	0.226	0.998
S380 0-15	853.765	844.857	3.342	0.093	0.997
S380 15-30	730.539	736.262	2.904	0.210	0.998

Overall, both bacterial richness and diversity exhibit more pronounced changes at lower altitudes compared to high altitudes. The Coverage index for all 24 sequencing samples exceeded 98%, indicating a high level of sample detection coverage. This underscores that the Alpha diversity index values effectively capture the true microbial composition in the sample.

### 3.5 Analysis of soil dominant bacteria

**Figure 2:**
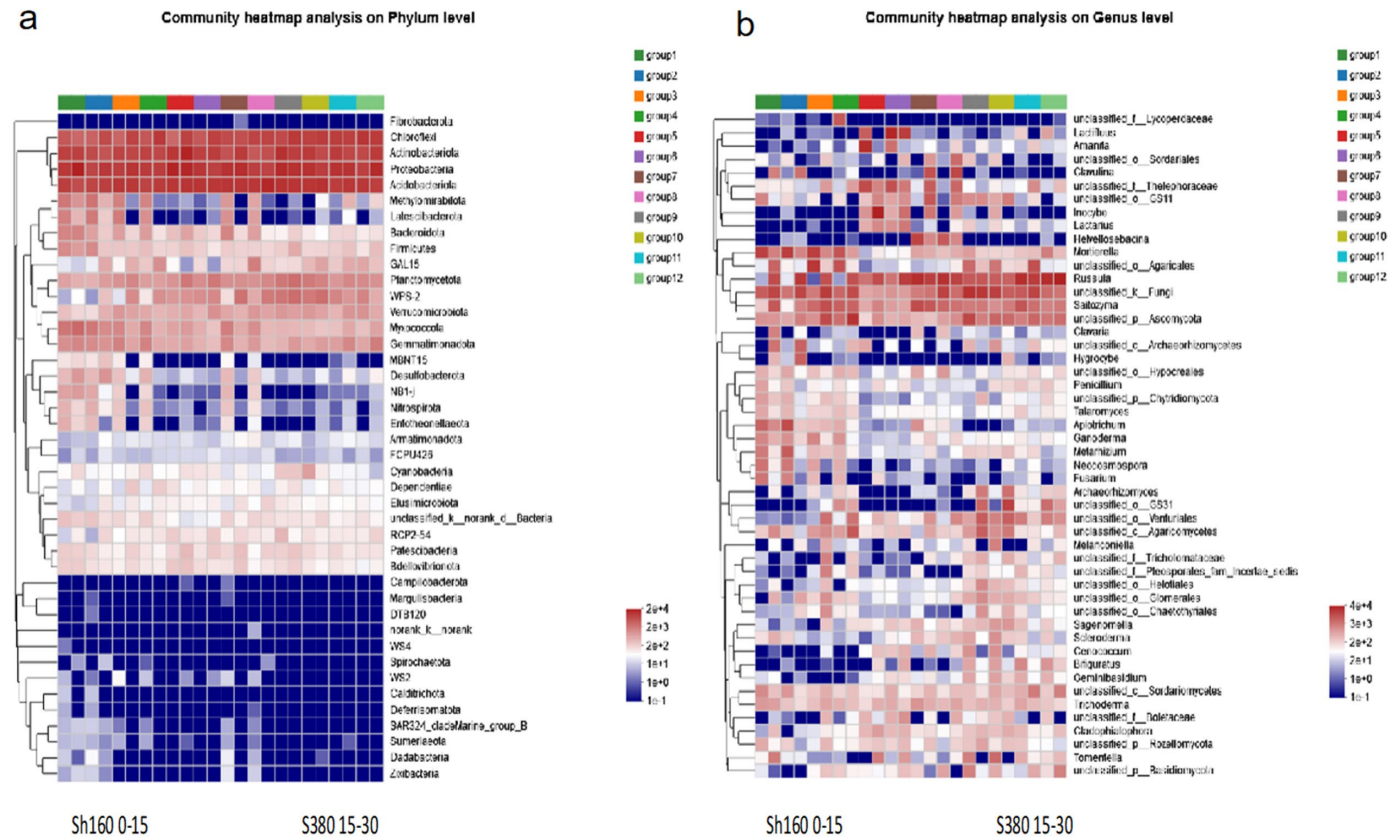
Structural changes of soli bacteria **(a)** and fungi **(b)**.

The taxonomic analysis results indicate that at the bacterial phylum level, the community structure composition of different groups is characterized by predominant bacterial phyla including Chloroflexi, Actinobacteriota, Proteobaceria, Acidobacteriota, Meltrytomirabitota, Latescibacterota, Planctomycetota, Verrucomicrobiota, Myxococcota, and Gemmatimonadota. Overall, the bacterial community richness is higher at lower altitudes compared to higher altitudes (Figure 2a). At the fungal genus level, the community structure analysis across different groups (Figure 2b) reveals a gradual decline in fungal richness with increasing altitude. Common dominant fungi identified include Lactfluus, Inocybe, Mortierella, Russula, Saitozyma, Apiotrichum, Ganoderma, Metarhizium, Sagenomelta, and Trichoderma.

**Figure 3:**
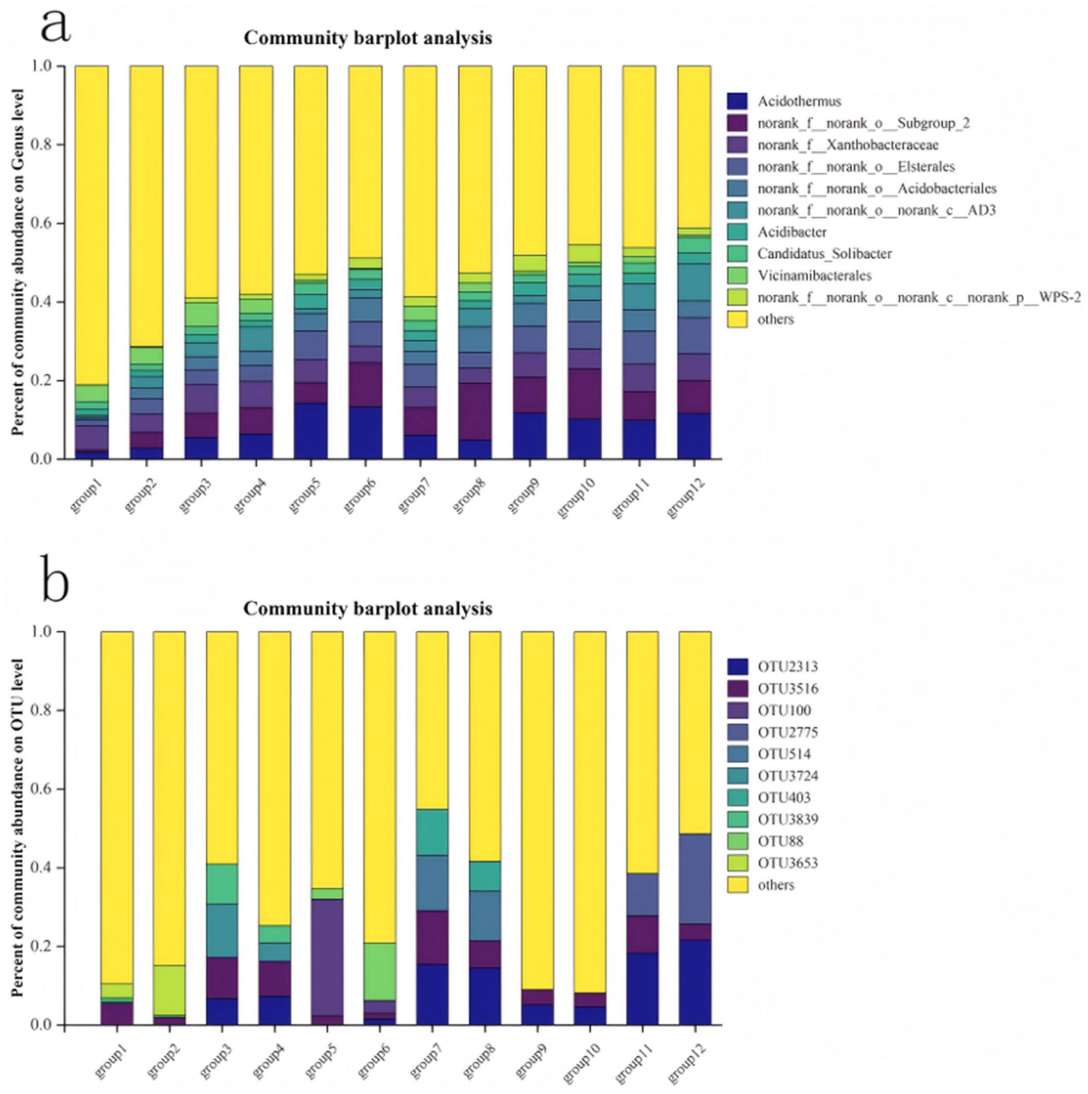
Structural changes of soli bacteria **(a)** and fungi OTU **(b)**.

**Figure 4:**
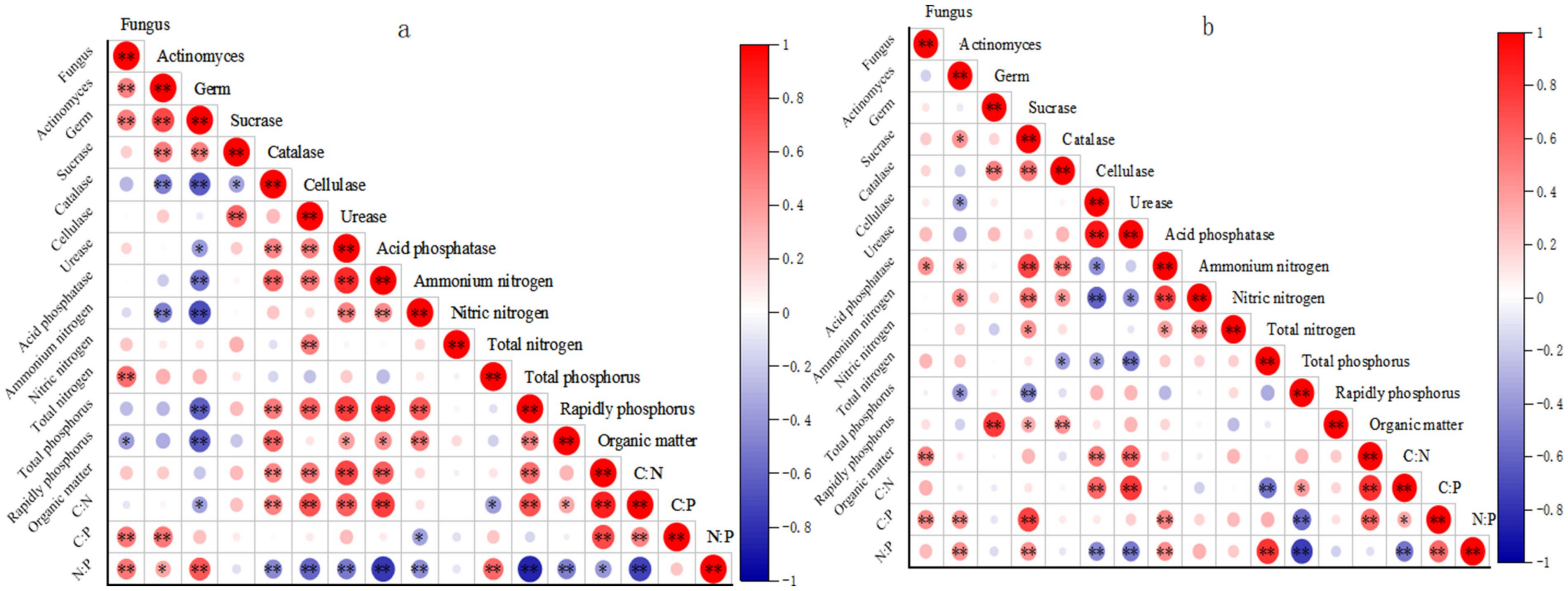
Correlation analysis results of soil indexes in shade slope **(a)** and sunny slope **(b)**. *means *p* < 0.05, a significant correlation; **means *p* < 0.01, a very significant correlation.

In Figure 3a, an insight is provided focusing into the community structure composition of different bacterial groups at the genus level. On the shady slope, there is a general increase in the total number of bacteria with rising altitude, whereas on the sunny slope, this trend is reversed compared to the shady slope. Each group exhibits dominant bacterial species, albeit with relatively consistent species numbers. Prominent species includ Acidothermus, norank_f_norank_o_Subgroup_2,norank f_ Xanthobacteraceae,norank_f_norank_o_Elsterales,norank_f_norank_o cidobacteriales, Candidatus_Solibacter, among others. The dominant fungal community structure is illustrated (Figure 3b). On shady slopes (Figure 4A), various soil indicators show significant correlations. The dominant fungi in this scenario include OTU3516 and OTU3839. Conversely, on sunny slopes, there is a gradual decrease followed by an increase in the total number of fungi, with dominant species identified as OTU2313 and OTU3516.

The principal component analysis aims to distill essential elements and structures, eliminate noise and redundancy, simplify the original data, and reveal underlying simple structures hidden within the complex dataset, as shown in Supplementary Figure 3. For bacteria, PC1 contributes 36.17% of the variance, while PC2 contributes 18.91%. Regarding fungi, PC1 accounts for 18.74% of the variance, while PC2 contributes 15%. The soil bacteria in the research area exhibited clustering into two distinct categories, whereas soil fungi displayed minimal differentiation, aligning with the hierarchical clustering analysis of the sample.

### 3.6 Culturable microorganisms at different altitudes

The composition and quantity of soil bacteria, actinomycetes, and fungi are shown in Supplementary Figure 4. The distribution of the bacteria populations at different altitudes exceeds that of fungi, with average values for bacteria and fungi amounting to 219166.667 and 8027.778, respectively, in the 0–15 cm soil layer. Across the altitude sampling points, the overall count of soil bacteria and fungi in the 0–15 cm soil layer surpassed that in the 15–30 cm soil layer. The count of culturable bacteria in the soil on shady slopes generally declines with increasing altitude (Supplementary Figure 4a). Additionally, the number of bacteria in the 0–15 cm soil layer surpasses that in the 15–30 cm soil layer. While there is an initial increase followed by a decrease in bacterial numbers, the disparity between the two soil layers is not pronounced. As depicted in Supplementary Figure 4b, the total count of fungi diminishes with altitude elevation, with the number of fungi in the 0–15 cm soil layer surpassing that in the 15–30 cm soil layer. Specifically, the fungal count in the 15–30 cm soil layer decreases with increasing altitude. The decline in fungal numbers in the 15–30 cm soil layer is more pronounced that in the 0–15 cm soil layer, with both reaching their lowest point at an altitude of 380 m.

### 3.7 Soil physical and chemical characteristics at different altitudes

With increasing altitude, the combined levels of ammonium nitrogen and nitrate nitrogen in both shady and sunny slope soils exhibit a pattern of initial increase followed by decrease (Supplementary Table 2). The total nitrogen and total phosphorus contents in the soil vary between shady and sunny slopes. Overall, there is a consistent upward trend in the available phosphorus content, with a gradual increase on shady slopes and an initial increase, followed by a decrease and subsequent increase on sunny slopes. Additionally, with increasing altitude, soil organic matter content demonstrates a gradual increase on shady slopes, while on sunny slopes, it displays an initial increase followed by a decrease.

On shady slopes, RCN and RCP exhibited an increasing-decreasing trend (Supplementary Figures 5a,b), while RCN on sunny slopes displayed a similar increasing-decreasing pattern (Supplementary Figure 5d). In contrast, RCP on sunny slopes demonstrated a decreasing trend with rising altitude (Supplementary Figure 5e). The RNP values for both shady and sunny slopes initially decreased, then increased, and finally decreased again as altitude increased (Supplementary Figures 5c,f).

### 3.8 Correlation analysis of soil parameters, redundancy analysis and equation structure model of soil nutrients, enzyme activities, and fungi

On shady slopes, various soil indicators show significant correlations. Invertase exhibits a notable negative correlation with available phosphorus, while catalase shows a significant positive correlation with organic matter. Cellulase demonstrates an extremely significant positive correlation with nitrate nitrogen and a significant positive correlation with organic matter. Additionally, there is a significant positive correlation between urease and nitrate nitrogen. An extremely significant positive correlation was observed between catalase and cellulase. Furthermore, an extremely significant positive correlation was found between acid phosphatase and invertase. Bacterial count exhibited an extremely significant positive correlation with organic matter, catalase, and urease, and a significant positive correlation with invertase. Fungi displayed an extremely significant positive correlation with invertase and urease.

The correlation among various indicators of soil on sunny slopes is displayed (Figure 4b). Catalase exhibits a significant positive correlation with organic matter. Soil cellulase demonstrates a highly significant positive correlation with nitrate nitrogen and organic matter. Soil urease shows a significant positive correlation with nitrate nitrogen, organic matter, and peroxidation. Hydrogenase and cellulase showed extremely significant positive correlation. A significant positive correlation was observed with nitrate nitrogen. Additionally, a significant positive correlation existed between acid phosphatase and invertase. A highly significant positive correlation was observed between bacterial count and organic matter, soil catalase, soil urease, and soil cellulase. Fungi displayed extremely significant positive correlations with soil invertase, acid phosphatase, urease, and bacterial count, while exhibiting significant negative correlations with total phosphorus.

**Figure 5:**
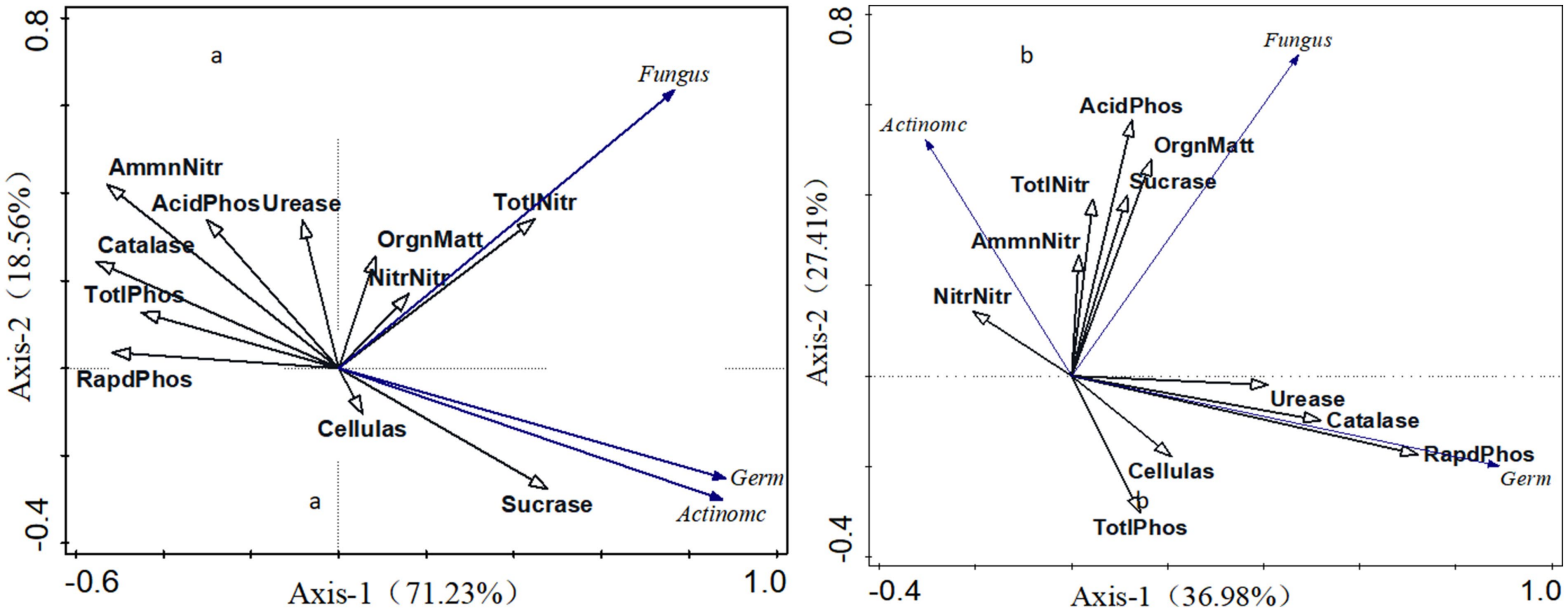
Redundancy analysis of soil nutrients, enzyme activities, and fungi on shady slopes **(a)** and sunny slopes **(b)**.

In this study, soil bacteria and fungi were selected as response factors, while soil nutrients and enzyme activities served as environmental factors for redundancy analysis, as depicted in Figure 5. The cumulative explanation rate of shady slope redundancy analysis reached 89.79%, with the first axis explaining 71.23% of the response variable and the second axis explaining 18.56%. Fungi are positively correlated with total nitrogen, nitrate nitrogen, organic matter, urease, and sucrase, with the strongest correlation observed with soil total nitrogen. Bacteria show positive correlations with sucrase, cellulase, total nitrogen, nitrate nitrogen, and organic matter, with sucrase exhibiting the most prominent association. The cumulative explanatory rate of the redundancy analysis for the Yang Slope reached 64.39%, with the first axis explaining 36.98% of the response variable and the second axis explaining 27.41%. Fungi exhibit positive correlations with enzyme activity, total nitrogen, available phosphorus, and organic matter, with the strongest correlation observed with organic matter. Bacteria show positive correlations with sucrase, acid phosphatase, total nitrogen, nitrate nitrogen, ammonium nitrogen, and organic matter, with sucrase being the most significant. Both fungi and bacteria in both shady and sunny slopes display positive correlations with sucrase, with the correlation between bacteria and sucrase being higher in shady slopes than in sunny slopes.

**Figure 6:**
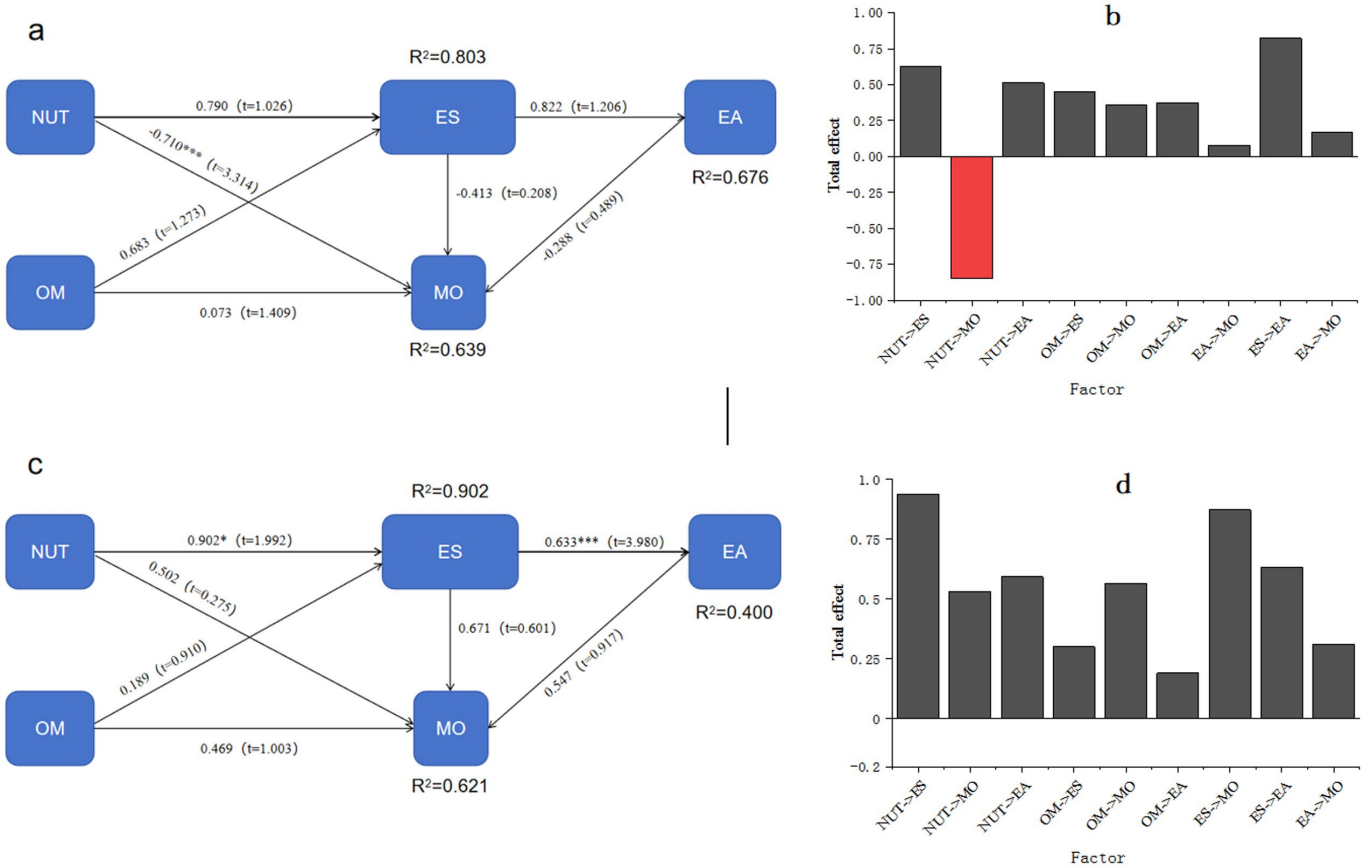
Equation structure model and total influence relationship for shady slopes **(a,b)** and sunny slopes **(c,d)**.

In this experiment, soil nutrients, enzyme activities, microbial community composition, ecological stoichiometry, and organic matter were selected for constructing a structural equation model (SEM), as shown in Figure 6. Notably, soil nutrients on shady slopes exhibited a significant negative correlation with fungi, with a correlation coefficient of −0.710. The analysis revealed that soil nutrients had the most substantial negative impact coefficient on microbial community composition, amounting to −0.851. Conversely, organic matter showed a positive correlation with microbial community composition and exerted a positive influence on the total fungal population. The most significant positive influence coefficient observed is 0.357. There is a positive correlation between soil nutrients and ecological stoichiometry, with a correlation coefficient of 0.790. The results show that soil nutrients have a more pronounced impact on ecological stoichiometry compared to organic matter. The cumulative influence coefficient of soil nutrients on enzyme activity is 0.513, and ecological stoichiometry displays a positive correlation with enzyme activity, with a correlation coefficient of 0.822. Among soil nutrients and organic matter, soil nutrients have a greater impact on ecological stoichiometry, underscoring their pivotal role in influencing enzyme activity. On sunny slopes, a positive correlation is observed between soil nutrients and microbial community composition, with a correlation coefficient of 0.502. The findings highlight that ecological stoichiometry yields the highest impact coefficient on microbial community composition, reaching 0.873. Additionally, there is a significant positive correlation between soil nutrients and ecological stoichiometry, with a correlation coefficient of 0.902. The results show that among soil nutrients and organic matter, soil nutrients have a greater impact on ecological stoichiometry. The cumulative influence coefficient of soil nutrients on enzyme activity amounts to 0.593, with ecological stoichiometry displaying a highly significant positive correlation with enzyme activity, represented by a correlation coefficient of 0.633. The influence pattern of soil nutrients and organic matter on ecological stoichiometry aligns with that observed in shady slopes.

## 4 Discussion

### 4.1 Effects of different altitudes on soil nutrients and ecological stoichiometry

Spatial variations in soil nutrients are evident across different altitudes. On shady slopes, there is an increasing trend in overall soil nutrient content with altitude. This trend aligns with the findings reported by Mbibueh et al. (2021) within a similar altitude range, indicating a consistent pattern. Consistent with studies on alpine meadows on the eastern Qinghai Tibet Plateau (Wang et al., 2023) and high-altitude areas in Yunnan (Wang et al., 2024), both indicate that elevation leads to organic matter accumulation. In contrast to the decreasing trend of soil nutrients with increasing altitude observed in the Loess Plateau (Li et al., 2024), the different rates of organic matter decomposition may be due to climate differences in the study area. The influence of enzyme activity is likely a contributing factor, where organic matter fosters favorable conditions for soil enzymes, thereby enhancing their stability and efficacy in driving the biological cycling of soil elements and organic matter, facilitating the release of essential nutrients. Higher enzyme activity correlates with faster nutrient transformation rates in the soil, ultimately enhancing plant nutrient uptake efficiency. In contrast, the total nitrogen content in soil on sunny slopes decreases with altitude, aligning with the findings of Lopez-Lopez et al. (2023). The excessive accumulation of organic matter causes high C/N, which limits the decomposition of organic matter by microorganisms. Microorganisms require nitrogen for synthesizing their cellular structure and metabolic products during the decomposition of organic matter.

The content and stoichiometric ratios of carbon, nitrogen, and phosphorus in soil play a crucial role in vegetation growth and soil nutrient cycling. This study found that the C/N ratio was high (shaded slope) and the N/P ratio was low (shaded slope), indicating nitrogen limitation, which is consistent with the research results in the Yunnan Karst areas (Wang, 2022). However, the C/N ratio on the sunny slope fluctuates greatly, which is different from the stable C/N trend (Bońska et al., 2025), possibly due to differences in organic matter mineralization rates caused by strong sunlight on the sunny slope. The C/N ratio (RCN) in soil serves as an indicator reflecting the decomposition rate of organic matter, with RCN being inversely related to the organic matter decomposition rate. A lower RCN signifies a faster mineralization rate of organic matter, leading to a higher nitrogen content. Conversely, a higher C/N ratio indicates slower mineralization and decomposition rates of organic matter, resulting in lower nitrogen content (Lorenzo, 2010). The observed trend of total nitrogen content in soil on sunny slopes with altitude contradicts the majority of researchers’ findings (Gelaw et al., 2014). This discrepancy may be attributed to the accumulation of organic matter increasing with higher altitude. Excessive organic matter content relative to total soil nitrogen leads to a high C/N ratio, restricting the release of mineralized nitrogen nutrients. Consequently, total nitrogen content at higher altitudes is lower than at low altitudes. The C/P ratio (RCP) in soil serves as an indicator of phosphorus availability, where a smaller RCP indicates higher phosphorus availability in the soil. This aligns with the observation that the available phosphorus content in soil at higher altitudes on shady slopes in this experiment exceeds that at low altitudes. The N/P ratio (RNP) in soils serves as a diagnostic indicator for nitrogen saturation and a threshold for identifying nutrient limitations. Both shady slopes and sunny slopes exhibit a similar trend in N/P ratios. A low N/P ratio indicates that nitrogen is the main limiting factor for crop growth.

### 4.2 Effects of different altitudes on soil enzyme activity and microbial population structure

Soil enzymes are important indicators for evaluating soil quality and microbial activity (Kögel-Knabner, 2009). The overall increase in soil acid phosphatase activity and cellulase activity with altitude is similar to the research results of Chang et al. (2016). Previous studies mainly discussed the factors that affect enzyme activity from the perspective of temperature and humidity. In contrast, this study focuses on the influence of organic matter and microbial activity, providing supplementary insights into the impact of altitude on enzyme activity. In the correlation analysis, organic matter content on shady slopes was positively correlated with soil enzyme activity, while the bacteria and fungi on sunny slopes were positively correlated with enzyme activity. The soil enzyme activity increased with altitude elevation. This pattern could be linked to the stimulation of microbial metabolic activity resulting from the rise in organic matter content, thereby indirectly enhancing soil enzyme activity (Zhang et al., 2016). These findings further support the significance of organic matter content and microorganisms as crucial factors affecting enzyme activity.

Alpha diversity analysis revealed that the richness and diversity of soil bacteria and fungi exhibited more pronounced changes at lower altitudes compared to higher altitudes, with distinct altitude-related characteristics observed in the microbial community structure overall. The decrease in bacterial diversity at higher altitudes can be attributed to the heightened sensitivity of bacteria to environmental variations such as temperature, which can limit their survival and reproductive capacities under challenging conditions at higher altitudes. This outcome differs from previous research findings, possibly due to the increase in relative abundance of fungi as altitude rises. Fungi rely on their low temperature tolerance and ability to decompose complex organic matter. While temperature typically serves as the primary controlling factor, this study specifically investigates altitude within the range of 160–380 m. In this context, where temperature variations have minimal impact, substantial changes in soil organic matter content occur, positively influencing fungi. Organic matter emerges as a key determinant influencing the survival, species composition, and metabolic activities of soil microorganisms (Sweeney et al., 2025). This alteration in the microbial community structure not only affects the path and rate of soil material recycling but also has the potential to alter the stability and functionality of the soil ecosystem.

The research subjects selected have a relatively small altitude span (160–380 meters) and lack a broader spectrum of altitude gradients and diverse climate zones. Meanwhile, the study area falls under a subtropical monsoon climate, potentially leading to significant differences in soil characteristics compared to regions like temperate and arid zones, thus constraining the generalizability of the findings. The study relies on single sampling data without accounting for the influences of seasonal fluctuations or long-term dynamics (such as interannual variations) on soil enzyme activity and microbial communities.

### 4.3 Key nutrients and enzyme factors affecting microbial population structure

In correlation analysis, redundancy analysis and SEM analysis, soil nutrients on shady slopes exhibited a negative correlation with microbial activity, whereas soil nutrients on sunny slopes demonstrated a positive correlation with microbial activity. These findings align with previous studies (Canfora et al., 2023; Bakker et al., 2015), underscoring that soil total nitrogen and organic matter content are the main factors affecting microbial communities. Additionally, available phosphorus plays a critical role in shaping soil microbial communities, driving microorganisms to adapt and optimize their phosphorus utilization efficiency. This evolutionary strategy selects for microorganisms that can efficiently utilize phosphorus, leading to shifts in population structure. Acid phosphatase facilitates the hydrolysis of organophosphorus compounds, converting organic phosphorus into inorganic phosphorus, thereby enhancing the available phosphorus content in the soil (Canfora et al., 2023). This study also revealed that catalase exerts a notable influence on available phosphorus and organic matter. This could be attributed to the bacterial growth environment necessitating soil nutrients for protection, thereby enhancing bacterial abundance and activity. Enzyme activity significantly impacts soil nutrients, contributing to the improvement of soil nutrient content through active participation in soil nutrient cycling (Xiao et al., 2024).

## 5 Conclusion

This study provides a comprehensive analysis of soil nutrients, soil enzyme activities, soil microbial characteristics, and their intricate interrelationships at low altitudes (160–380 m). The research findings are as follows:

With increasing altitude, soil nutrients on shady slopes tend to rise, while the changes in various indicators on sunny slopes with altitude are less pronounced. Nitrogen levels in the study area are low, with nitrogen being the main influence factor. In response to varying altitudes, the richness of soil bacteria and fungi on shady slopes exhibited higher levels at lower altitudes compared to higher altitudes. As altitude increased, bacterial richness on sunny slopes initially decreased before rising again. Conversely, on shady slopes, bacterial numbers increased with altitude, while fungal numbers displayed an initial increase followed by a decrease, in contrast to the trend observed on sunny slopes. Soil organic matter content emerged as the predominant factor influencing enzyme activity, with key nutrient factors affecting microorganisms encompassing total nitrogen content, organic matter content, and available phosphorus content.

The findings from this study offer a theoretical foundation for enhancing soil fertility and managing soil ecosystem health. Nonetheless, this study was limited to low-altitude conditions. Future research endeavors should broaden the scope to encompass diverse ecological regions, thereby furnishing more comprehensive data to guide crop cultivation, land management, and enhancement strategies in southwestern China.

## Data Availability

The original contributions presented in the study are included in the article/Supplementary material, further inquiries can be directed to the corresponding authors.

## References

[ref2] Bakker M. G. Chaparro J. M. Manter D. K. Vivanco J. M. (2015). Impacts of bulk soil microbial community structure on rhizosphere microbiomes of *Zea mays*. Plant Soil 392, 115–126. doi: 10.1007/s11104-015-2446-0

[ref3] Bońska E. Lasota J. Prauch W. Ilek A. (2025). Vertical variations in enzymatic activity and C: N: P stoichiometry in forest soils under the influence of different tree species. Eur. J. For. Res. 144, 83–94. doi: 10.1007/s10342-024-01742-5

[ref4] Canfora L. Tartanus M. Manfredini A. Tkaczuk C. Majchrowska-Safaryan A. Malusà E. (2023). The impact of Beauveria species bioinocula on the soil microbial community structure in organic strawberry plantations. Front. Microbiol. 13:13. doi: 10.3389/fmicb.2022.1073386, PMID: 36713158 PMC9874679

[ref5] Chang E. H. Chen T. H. Tian G. Chiu C. Y. (2016). The effect of altitudinal gradient on soil microbial community activity and structure in moso bamboo plantations. Appl. Soil Ecol. 98, 213–220. doi: 10.1016/j.apsoil.2015.10.018, PMID: 40166663

[ref6] Chen S. Zhou Y. Chen Y. Gu J. (2018). Fastp: an ultra-fast all-in-one FASTQ preprocessor. Bioinformatics 34, i884–i890. doi: 10.1093/bioinformatics/bty560, PMID: 30423086 PMC6129281

[ref7] Delgado-Baquerizo M. (2019). Obscure soil microbes and where to find them. ISME J. 13, 2120–2124. doi: 10.1038/s41396-019-0405-0 30926921 PMC6776042

[ref8] Dietert R. R. Dietert J. M. (2024). Examining sound, light, and vibrations as tools to manage microbes and support Holobionts, ecosystems, and technologies. Microorganisms 12:905. doi: 10.3390/microorganisms12050905 38792734 PMC11123986

[ref9] Fuentes B. Gómez F. Valdez C. Videla A. Castro-Severyn J. Barahona S.. (2022). Effects of altitude on soil properties in coastal fog ecosystems in Morro Moreno National Park, Antofagasta, Chile. Eur. J. Soil Sci. 73:e13217. doi: 10.1111/ejss.13217

[ref10] Gelaw A. M. Singh B. R. Lal R. (2014). Soil organic carbon and total nitrogen stocks under different land uses in a semi-arid watershed in Tigray, Northern Ethiopia. Agricult. Ecosyst. Environ. 188, 256–263. doi: 10.1016/j.agee.2014.02.035

[ref11] Guan S. Y. (1986). Soil enzymes and their research methods. Beijing: Agriculture Press.

[ref12] Guan S. Y. Meng Z. P. (1986). Changes of agricultural properties and enzyme activities of black soil with different reclamation years. Chin. J. Soil Sci. 4, 157–159.

[ref13] Holguin G. Vazquez P. Bashan Y. (2001). The role of sediment microorganisms in the productivity, conservation, and rehabilitation of mangrove ecosystems: an overview. Biol. Fertil. Soils 33, 265–278. doi: 10.1007/s003740000319

[ref14] Jacobson Meyers M. E. Sylvan J. B. Edwards K. J. (2014). Extracellular enzyme activity and microbial diversity measured on seafloor exposed basalts from Loihi seamount indicate the importance of basalts to global biogeochemical cycling. Appl. Environ. Microbiol. 80, 4854–4864. doi: 10.1128/AEM.01038-14, PMID: 24907315 PMC4135773

[ref15] Kögel-Knabner K. S. (2009). Alteration of soil organic matter pools and aggregation in semi-arid steppe topsoils as driven by organic matter input. Eur. J. Soil Sci. 60, 198–212. doi: 10.1111/j.1365-2389.2008.01104.x

[ref16] Kumar S. Garkoti S. C. (2022). Rhizosphere influence on soil microbial biomass and enzyme activity in banj oak, chir pine and banj oak regeneration forests in the central Himalaya. Geoderma 409:115626. doi: 10.1016/j.geoderma.2021.115626, PMID: 40166663

[ref7001] Li Q. Zhang Y. Li X. Liu X. Zhang D. (2024). Response of soil nutrients and enzyme activities to altitude in the grassland in the central Qilian Mountains. EGrassland and Turf 44, 26–33.

[ref17] Liu B. R. (2010). Changes in soil microbial biomass carbon and nitrogen under typical plant communies along an altitudinal gradient in east side of Helan Mountain. Ecology and Environmental Sciences. doi: 10.3724/SP.J.1035.2010.01150

[ref18] Liu C. Zhao D. Ma W. Guo Y. Wang A. Wang Q.. (2016). Denitrifying sulfide removal process on high-salinity wastewaters in the presence of *Halomonas* sp. Appl. Microbiol. Biotechnol. 100, 1421–1426. doi: 10.1007/s00253-015-7039-6, PMID: 26454867

[ref19] Lopez-Lopez A. B. Vazquez-Selem L. Siebe C. Cruz-Flores G. Correa-Metrio A. (2023). Effect of elevation and slope orientation on pedogenesis of late Holocene volcanic ash on a tropical high mountain in Central Mexico. CATENA 231:107288. doi: 10.1016/j.catena.2023.107288, PMID: 40166663

[ref20] Lorenzo P. (2010). Susana Rodríguez-Echeverría, Luís González, et al. effect of invasive *Acacia dealbata* link on soil microorganisms as determined by PCR-DGGE. Appl. Soil Ecol. 44, 245–251. doi: 10.1016/j.apsoil.2010.01.001

[ref21] Magoč T. Salzberg S. L. (2011). FLASH: fast length adjustment of short reads to improve genome assemblies. Bioinformatics 27, 2957–2963. doi: 10.1093/bioinformatics/btr507, PMID: 21903629 PMC3198573

[ref22] Mbibueh B. T. Fokeng R. M. Tume S. J. P. (2021). Effects of land cover/use change and altitude on soil NPK nutrients in selected areas in the north west region of Cameroon. Adv. Environ. Eng. Res. 2:1. doi: 10.21926/aeer.2104038

[ref23] Meng F. Q. Peng P. C. Meng X. L. Li H. Liu Z. (2022). Characteristics and formation conditions of landslide geological hazards in Youjiang District, Baise City, Guangxi. Resour. Informat. Eng. 37, 41–43+46. doi: 10.19534/j.cnki.zyxxygc.2022.03.036

[ref24] Obayomi O. Taggart C. B. Zeng S. Sefcik K. Willis B. Muir J. P.. (2023). Dairy manure-derived biochar in soil enhances nutrient metabolism and soil fertility, altering the soil prokaryote community. Agronomy 13:1512. doi: 10.3390/agronomy13061512, PMID: 40053772

[ref25] Sainju U. M. Liptzin D. Dangi S. M. (2022). Enzyme activities as soil health indicators in relation to soil characteristics and crop production. Agrosyst. Geosci. Environ. 5:10.1002/agg2.20297. doi: 10.1002/agg2.20297, PMID: 40165004

[ref26] Schinner F. (1982). Soil microbial activities and litter decomposition related to altitude. Plant Soil 65, 87–94. doi: 10.1007/BF02376806

[ref27] Shah T. Khan Z. Asad M. Imran A. Niazi M. B. K. Dewil R.. (2024). Straw incorporation into microplastic-contaminated soil can reduce greenhouse gas emissions by enhancing soil enzyme activities and microbial community structure. J. Environ. Manag. 351:351. doi: 10.1016/j.jenvman.2023.119616, PMID: 38042071

[ref28] Sweeney C. J. Melanie B. Rishabh K. Aderjan E. Sherborne N. (2025). Functional versus compositional tests in the risk assessment of the impacts of pesticides on the soil microbiome. Environ. Toxicol. Chem. 44, 1120–1133. doi: 10.1093/etojnl/vgaf012 39987504

[ref7002] Wan H. Chen L. Pang D. Ma J. Chen G. Li X. (2021). Soil enzyme activities and their stoichiometry at different altitudes in Helan Mountains, Northwest China. Journal of Applied Ecology. 32, 3045–3052. 34658188 10.13287/j.1001-9332.202109.021

[ref29] Wang J. Y. (2022). Prediction of ecosystem versatility by soil microbial community network complexity at different elevations in Qinghai-Tibet alpine meadow. Xianyang: Northwest A & F University.

[ref30] Wang J. Cai Q. (2024). Nitrogen migration and transformation characteristics of the soil in karst areas under the combined application of oxalic acid and urea inhibitors. Front. Plant Sci. 15:1386912. doi: 10.3389/fpls.2024.1386912, PMID: 38817941 PMC11137297

[ref31] Wang S. Du T. Liu S. Ma Y. Luo L. Zhu W.. (2023). Nature-derived hollow Micron-tubular signal tracers conquering the size limitations for multimodal Immunochromatographic detection of antibiotics. Anal. Chem. 95, 16958–16966. doi: 10.1021/acs.analchem.3c03230, PMID: 37942854

[ref32] Wang S. Li Q. Ye C. Ma W. Sun Y. Zhao B.. (2024). Eects of mulch films with dierent thicknesses on the microbial community of tobacco rhizosphere soil in Yunnan laterite. Front. Microbiol. 15:1458470. doi: 10.3389/fmicb.2024.1458470, PMID: 39376702 PMC11456438

[ref33] Wu J. S. (2006). Soil microbial biomass determination method and its application. Beijing: China Meteorological Press.

[ref1] Xiao S. Li B. Zhang M.. (2024). Effects of different tillage measures combined with straw returning on soil enzyme activity and microbial community structure and diversity. Agriculture. 15. doi: 10.3390/agriculture15010056

[ref34] Xie X. M. (2014). Soil and plant nutrition [M]. Hangzhou: Zhejiang University Publishing.

[ref35] Yokamo S. Irfan M. Huan W. Wang B. Wang Y. Ishfaq M.. (2023). Global evaluation of key factors influencing nitrogen fertilization efficiency in wheat: a recent meta-analysis (2000-2022). Front. Plant Sci. 14:1272098. doi: 10.3389/fpls.2023.1272098, PMID: 37965011 PMC10642427

[ref36] Zhang Z. Hu L. Liu Y. Guo Y. Tang S. Ren J. (2025). Land use shapes the microbial community structure by altering soil aggregates and dissolved organic matter components. J. Integr. Agricult. 24, 827–844. doi: 10.1016/j.jia.2024.07.018, PMID: 40166663

[ref37] Zhang H. Shi L. Wen D. Yu K. (2016). Soil potential labile but not occluded phosphorus forms increase with forest succession. Biol. Fertil. Soils 52, 41–51. doi: 10.1007/s00374-015-1053-9

