# Supplementary material for: Response of microbial structure characteristics and enzyme activity to different altitude
Source: Front Microbiol. 2025 Apr 23;16:1588591. doi: 10.3389/fmicb.2025.1588591 (PMC12055782; doi:10.3389/fmicb.2025.1588591)
Supplement: Supplementary file 1 [file Data_Sheet_1.pdf]

Response of microbial structure characteristics and enzyme activity to different altitude

Qiu-liang CAI [a1], Gui-kang JIA[a1*],Chun WEI [a],Ning ZHONG [b*],Ling-ling LV [a],Jian LI [a],Hong PANG [a],Wei YANG [a]

a.Guangxi Key Laboratory of Biology for Mango, Agriculture and Food Engineering College, Baise University， Industrial College of Subtropical Characteristic Agriculture，Baise，Guangxi 533000；

b.Minnan Normal University，Fujian Zhangzhou 363000

List of Table

Table SI1 Soil sample information

Table SI2 Soil nutrient characteristics at different elevations

List of Figures

Fig. SI1 Sparse curves of soil bacteria (a) and fungi (b)

Fig. SI2 Venn analysis of soil bacteria (a) and fungi (b) OTUs

Fig. SI3 Principal component analysis of soil bacteria (a) and fungi (b)

Fig. SI4 Distribution characteristics of soil bacteria(a) and fungi (b) at different altitudes

Fig. SI5 Soil ecological stoichiometric ratios c/n (a), c/p (b), n/p (c) on shady slopes, c/n (d), c/p(e), n/p (f) on sunny slopes at different altitudes.

Table SI1 Soil sample information

slope surface	Altitude(m)	longitude and latitude	sample name (0-15 cm)	sample name (15-30 cm)
shady slope	160	E106°38′42″N23°57′48″	Sh160 0-15	Sh160 15-30
	190	E106°38′42″N23°57′40″	Sh190 0-15	Sh190 15-30
	220	E106°38′42″N23°57′32″	Sh220 0-15	Sh220 15-30
	250	E106°38′42″N23°57′29″	Sh250 0-15	Sh250 15-30
	300	E106°38′42″N23°57′24″	Sh300 0-15	Sh300 15-30
	380	E106°38′42″N23°57′23″	Sh380 0-15	Sh380 15-30
sunny slope	160	E106°38′42″N23°57′20″	S160 0-15	S160 15-30
	190	E106°38′42″N23°57′18″	S190 0-15	S190 15-30
	220	E106°38′42″N23°57′16″	S220 0-15	S220 15-30
	250	E106°38′42″N23°57′14″	S250 0-15	S250 15-30
	300	E106°38′42″N23°57′10″	S300 0-15	S300 15-30
	380	E106°38′42″N23°57′05″	S380 0-15	S380 15-30

Table SI2 Soil nutrient characteristics at different elevations

number	NH^{4+}-N	NO^{-3}-N	N	P	A-P	Organic matter	number	NH^{4+}-N	NO^{-3}-N	N	P	A-P	Organic matter
Sh160 0-15	0.023±0.003cd	0.016±0.007 bc	0.124±0.004ab	20.746±0.51e	138.158±66.3ab	34.32±1.03b	S160 0-15	0.026±0.006 ab	0.028±0.004 abc	0.197±0.014ab	21.56±1.49d	77.4±38.7ab	38.86±3.10 d
Sh190 0-15	0.027±0.005 cd	0.035±0.010 ab	0.15±0.053 a	23.799±0.21 d	105±36.84 ab	37.83±2.06 e	S190 0-15	0.044±0.006 a	0.021±0.004 abc	0.191±0.048 a	16.629±1.50 e	93.9±51.6ab	44.70±2.75 c
Sh220 0-15	0.028±0.003cd	0.037±0.003 ab	0.161±0.006) b	26.673±0.60 c	103.158±42.37ab	53.65±0.69c	S220 0-15	0.034±0.002 ab	0.014±0.002 bc	0.137±0.003 b	22.576±0.88 d	182.4±18.4a	57.09±2.06 b
Sh250 0-15	0.043±0.03 a	0.051±0.009 a	0.173±0.004b	27.867±0.73 b	217.368±23.95 b	55.74±2.41 d	S250 0-15	0.021±0.050 b	0.014±0.002 ab	0.145±0.007 b	25.994±1.34 a	121.6±49.7b	59.50±0.34 b
Sh300 0-15	0.04±0.001 a	0.029±0.001 ab	0.173±0.052 ab	24.438±0.64 d	125.263±27.63 ab	67.07±1.72 b	S300 0-15	0.021±0.008 b	0.034±0.001 abc	0.122±0.004 ab	16.793±0.98 c	169.5±16.6ab	61.91±0.69 a
Sh380 0-15	0.04±0.012 ab	0.017±0.005 c	0.174±0.004 ab	35.37±1.03 a	173.158±16.58 ab	80.14±4.47 a	S380 0-15	0.022±0.008 b	0.021±0.005 abc	0.108±0.058 ab	14.928±0.82 b	187.9±64.5ab	47.46±2.75 c
Sh160 15-30	0.021±0.002 cd	0.019±0.009 bc	0.114±0.01 ab	19.962±0.69d	58.026±29.47 a	31.62±1.03 b	S160 15-30	0.02±0.004 b	0.01±0.002 bc	0.233±0.054 a	17.084±1.75 c	103.2±68.2 ab	30.27±2.75 d
Sh190 15-30	0.021±0.006 cd	0.023±0.003 bc	0.128±0.006 b	13.828±0.37 e	108.684±42.37 ab	35.36±1.03 d	S190 15-30	0.039±0.010 a	0.012±0.003 bc	0.136±0.006 c	21.308±1.10b	189.7±42.4ab	28.90±2.00 c
Sh220 15-30	0.028±0.005 ab	0.019±0.009 bc	0.161±0.048 ab	22.963±0.46c	149.211±1.84ab	45.74±0.34 a	S220 15-30	0.029±0.004 a	0.013±0.013 c	0.144±0.015 ab	25.541±1.24 c	224.7±47.9 ab	47.76±2.35 b
Sh250 15-30	0.048±0.005 bcd	0.015±0.006 c	0.161±0.003 a	26.464±0.22 b	145.526±53.42 ab	46.80±0.34 c	S250 15-30	0.035±0.002 a	0.040±0.003 ab	0.135±0.043 bc	20.048±2.49 a	81.1±25.8ab	55.37±0.34 a
Sh300 15-30	0.035±0.007 d	0.02±0.001 bc	0.125±0.006 b	26.791±0.98 b	184.211±47.89 ab	48.40±0.69 a	S300 15-30	0.02±0.008 b	0.018±0.008 bc	0.106±0.004 c	14.195±0.37 b	95.8±33.2b	33.36±1.03 d
Sh380 15-30	0.038±0.006 cd	0.019±0.009 bc	0.122±0.042 b	31.874±0.61 a	114.211±53.42 ab	53.34±2.06 b	S380 15-30	0.016±0.003b	0.011±0.001 bc	0.090±0.007 c	12.581±0.60 a	101.3±35.0 a	29.92±4.47 d

Note: Average ± standard deviation is listed, and different letters after the same column of data indicate significant differences ($P<0.05$).

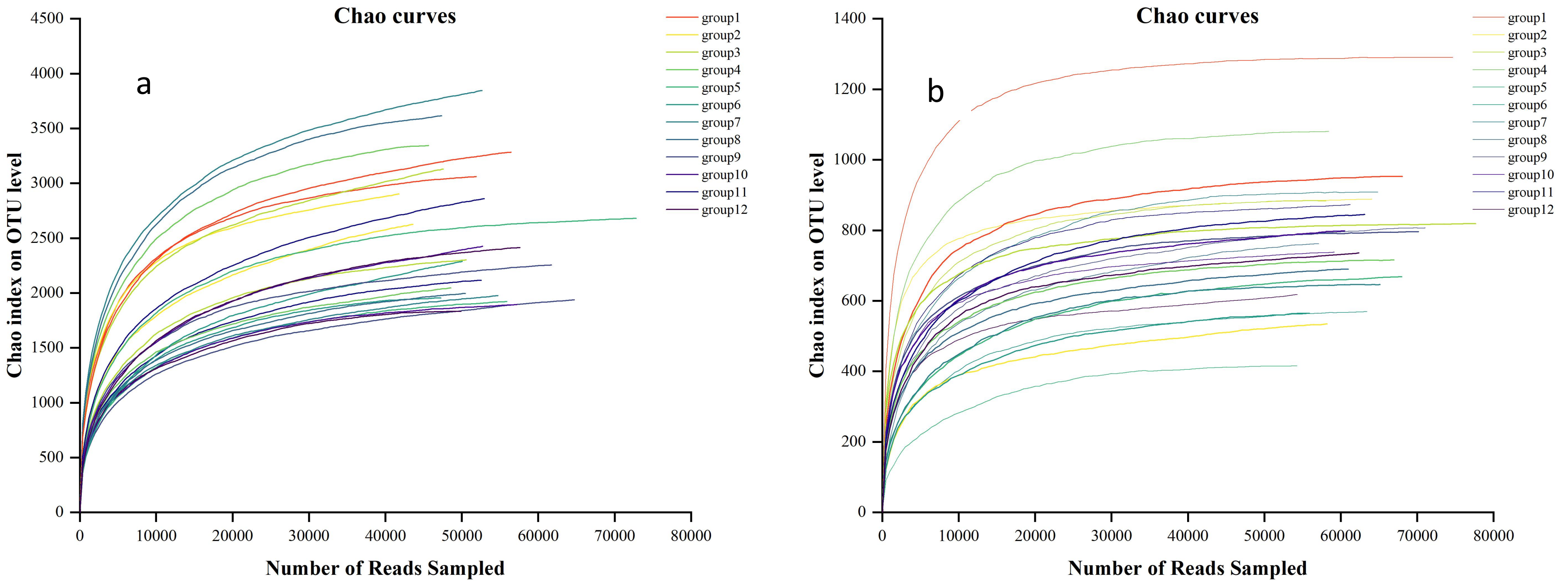

Fig. SI1 Sparse curves of soil bacteria (a) and fungi (b)

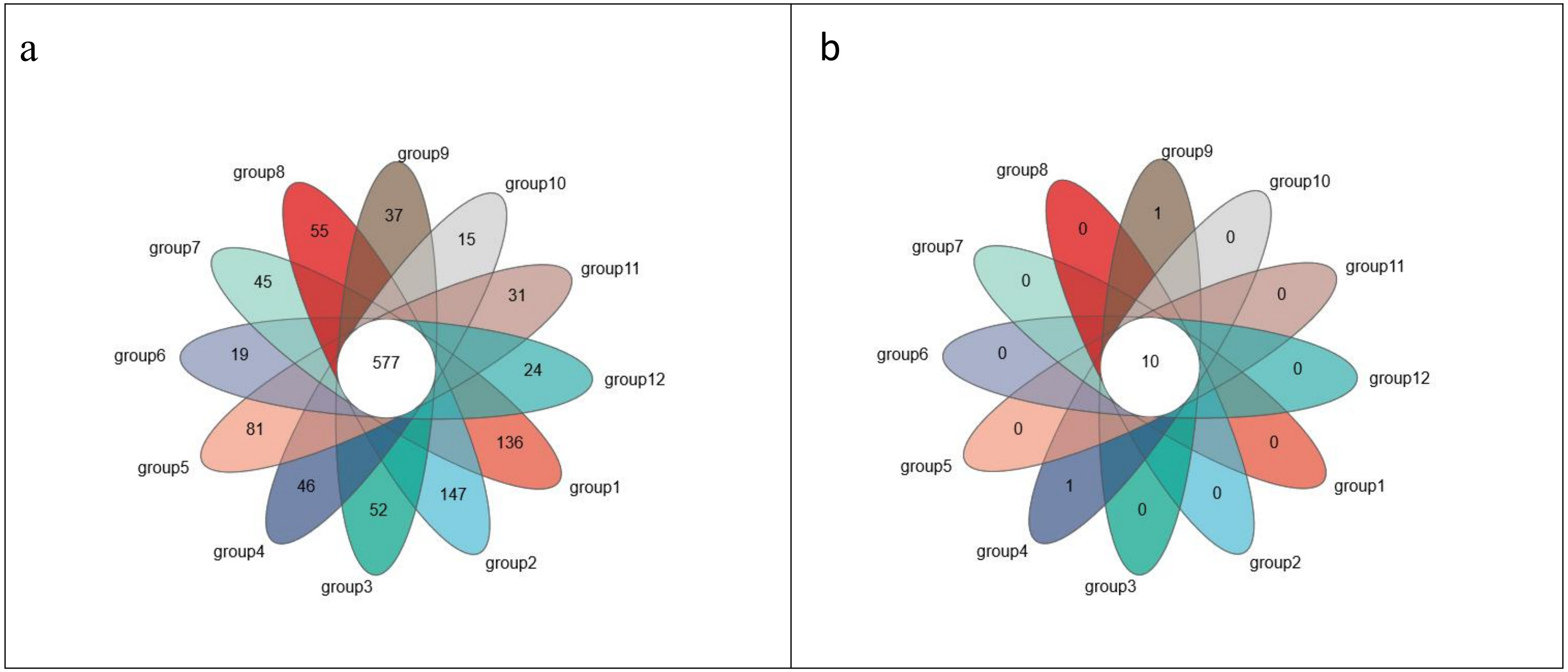

Fig.SI2 Venn analysis of soil bacteria (a) and fungi (b) OTUs

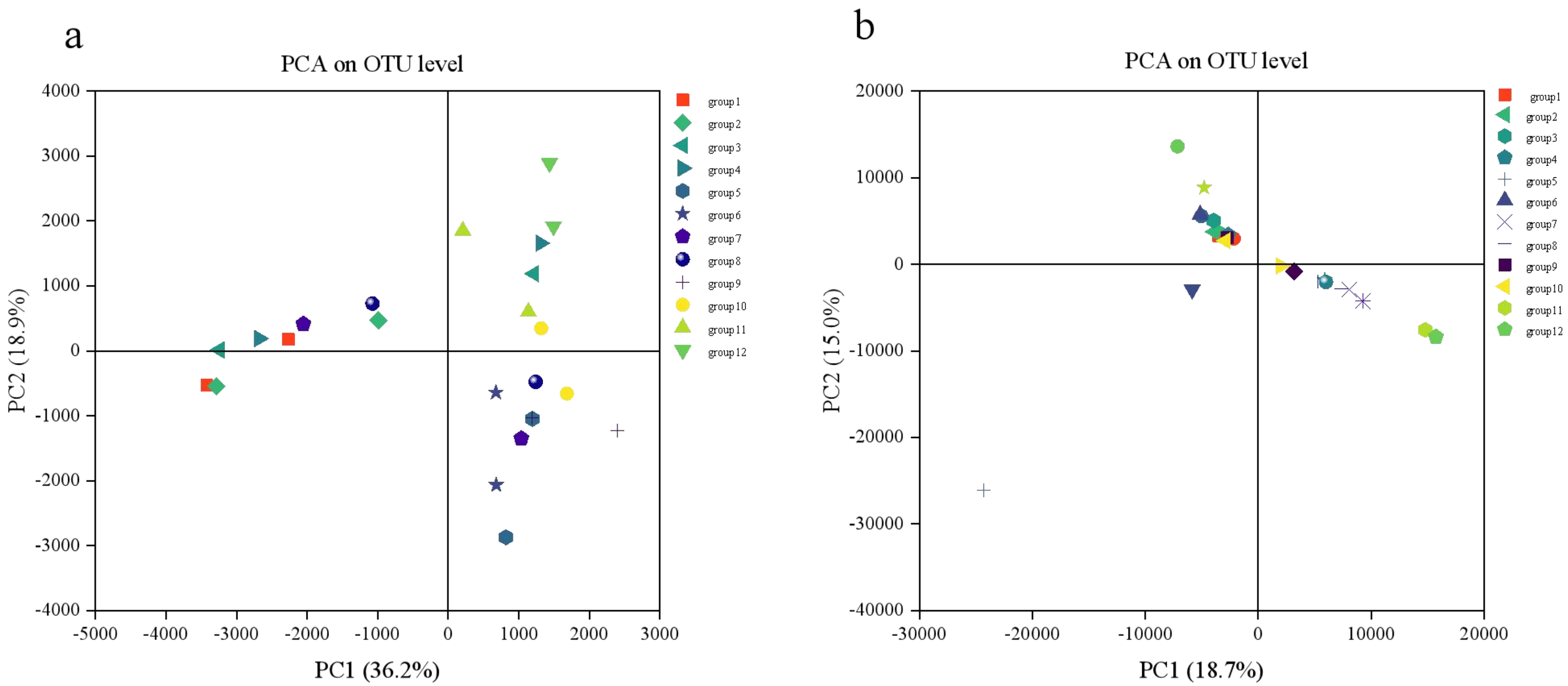

Fig. SI3 Principal component analysis of soil bacteria (a) and fungi (b)

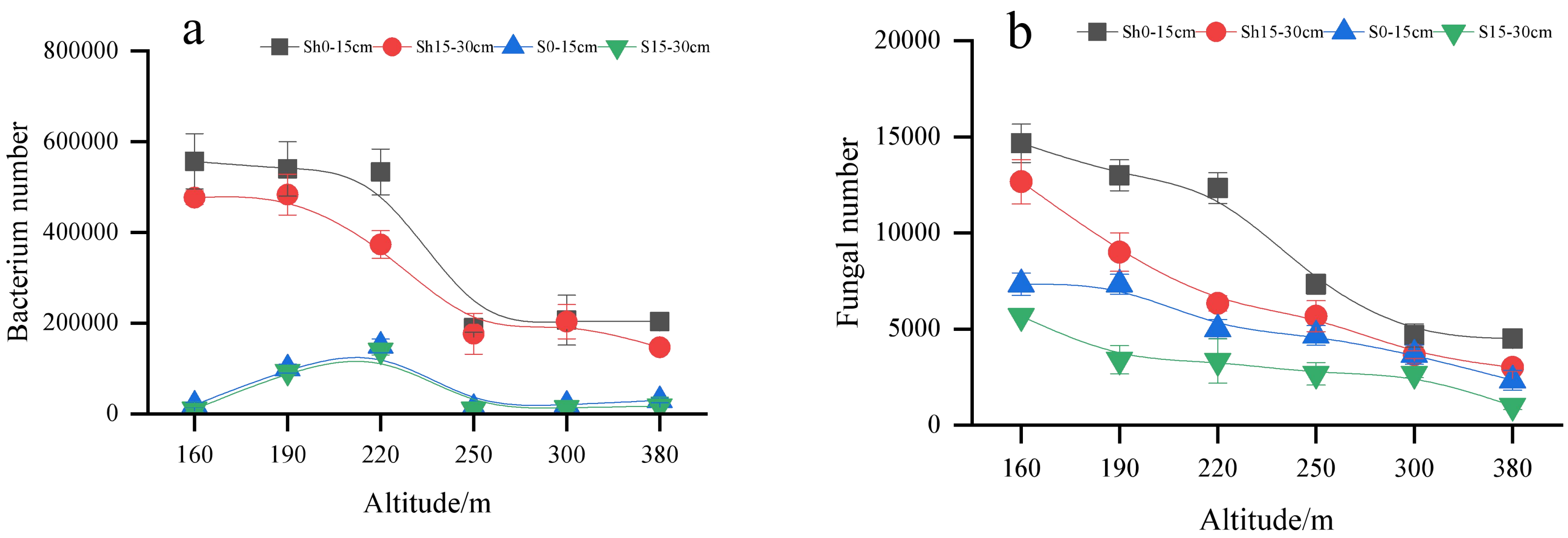

Fig. SI4 Distribution characteristics of soil bacteria(a) and fungi (b) at different altitudes

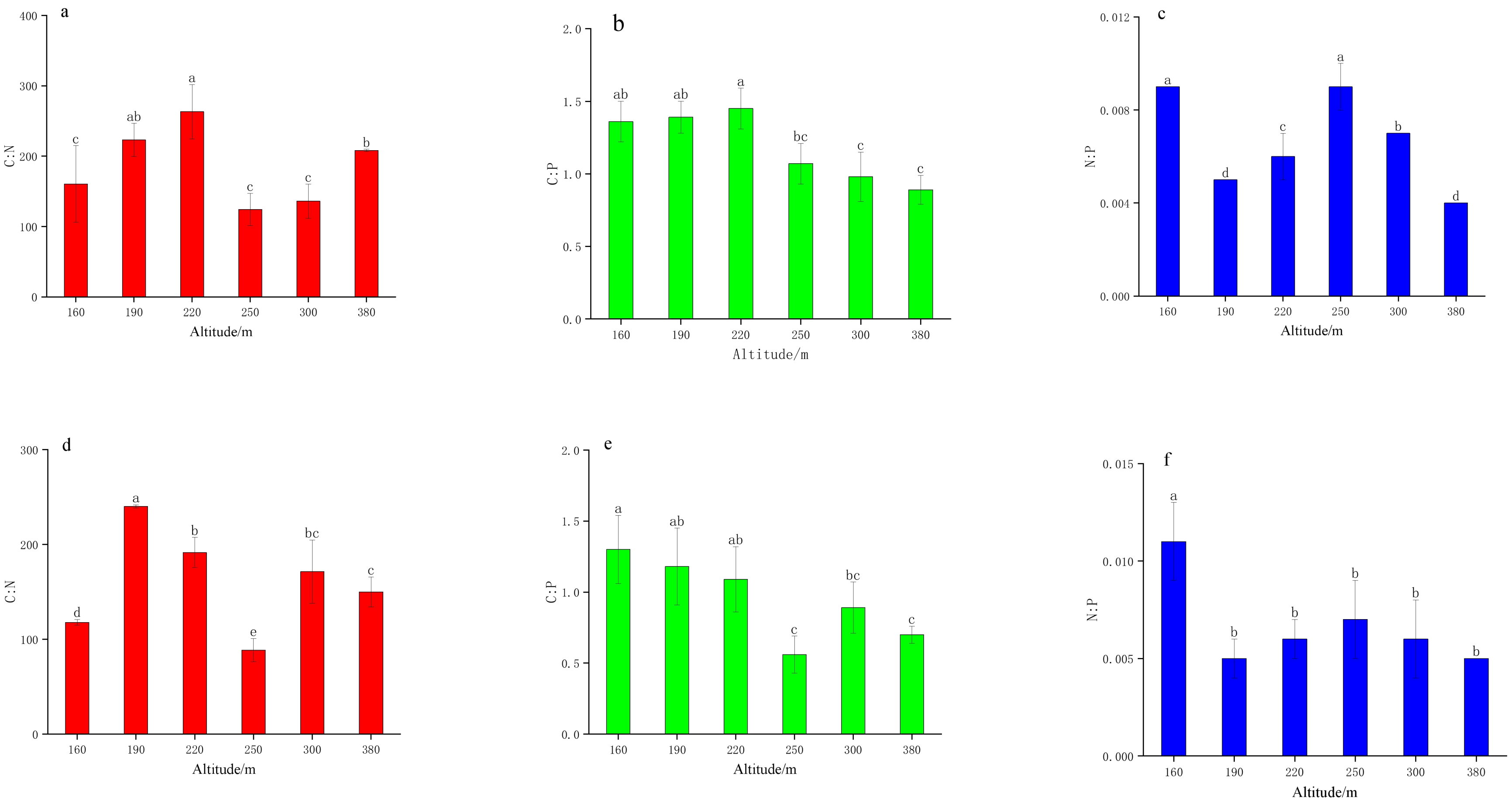

Fig. SI5 Soil ecological stoichiometric ratios c/n (a), c/p (b), n/p (c) on shady slopes, c/n (d), c/p, (e), n/p (f) on sunny slopes at different altitudes.

Note: c, n, and p are the average values of organic carbon (organic matter divided by 1.724), total nitrogen, and total phosphorus in the topsoil layer 0-30, respectively.
